# Multi-task deep learning for cardiac rhythm detection in wearable devices

**DOI:** 10.1038/s41746-020-00320-4

**Published:** 2020-09-09

**Authors:** Jessica Torres-Soto, Euan A. Ashley

**Affiliations:** 1 Department of Biomedical Informatics, Stanford University, Stanford, CA USA; 2 Department of Medicine, Division of Cardiovascular Medicine, Stanford University, Stanford, CA USA

**Keywords:** Atrial fibrillation, Biomedical engineering

## Abstract

Wearable devices enable theoretically continuous, longitudinal monitoring of physiological measurements such as step count, energy expenditure, and heart rate. Although the classification of abnormal cardiac rhythms such as atrial fibrillation from wearable devices has great potential, commercial algorithms remain proprietary and tend to focus on heart rate variability derived from green spectrum LED sensors placed on the wrist, where noise remains an unsolved problem. Here we develop DeepBeat, a multitask deep learning method to jointly assess signal quality and arrhythmia event detection in wearable photoplethysmography devices for real-time detection of atrial fibrillation. The model is trained on approximately one million simulated unlabeled physiological signals and fine-tuned on a curated dataset of over 500 K labeled signals from over 100 individuals from 3 different wearable devices. We demonstrate that, in comparison with a single-task model, our architecture using unsupervised transfer learning through convolutional denoising autoencoders dramatically improves the performance of atrial fibrillation detection from a F1 score of 0.54 to 0.96. We also include in our evaluation a prospectively derived replication cohort of ambulatory participants where the algorithm performed with high sensitivity (0.98), specificity (0.99), and F1 score (0.93). We show that two-stage training can help address the unbalanced data problem common to biomedical applications, where large-scale well-annotated datasets are hard to generate due to the expense of manual annotation, data acquisition, and participant privacy.

## Introduction

Wearable devices are increasingly used in cardiology for out-of-the-clinic healthcare monitoring^1^. The use of wearable devices in physical activity applications has allowed measurements of physiological parameters like ECG, heart rate, heart rhythm, to occur with greater frequency, convenience, and improved accuracy^2,3^. The fast expansion of these wearable device functionalities in a healthcare setting can help engage individuals in understanding disease progression and may allow for the detection of early disease trajectories^1^. Wrist-based smartwatch sensing for healthcare has generally been focused on photoplethysmography (PPG) for the detection of heart rate and heart rhythm. Of the abnormal heart rhythms, atrial fibrillation (AF), characterized by a disorganized chaotic electrical activity of the atria, has received the most attention. The incentive for studying AF detection methods in wearable devices is substantial. AF can often go unnoticed and yet is a risk factor for stroke; early AF detection may allow interventions that could decrease stroke risk. In addition, in the decades to come, the aging population will lead to a doubling in the prevalence of AF. There will be an increasing public health need for cost-effective methods for AF detection and monitoring to prevent disparities in care.

Given this potential for wearable devices to impact AF, correctly estimating and managing signal input quality is critical for AF detection methods. Historically, event detection for abnormal cardiac events relied on explicit rules and domain expert knowledge to craft features with high discriminatory power. However, noise in wrist-worn wearable devices remains an unsolved problem^4^. Inaccurate heart rate estimation and misdetection of AF are largely caused by poor signal quality^5,6^. Recent work in developing an optimal signal quality index (SQI) relies on manually selected features to try to distinguish high-quality PPG signals from poor or corrupted signals^7,8^. The limitations of manually selected features include the challenge of designing consistent descriptors/features for diverse PPG environments, across different individuals, while maintaining high discriminatory power^7^. Published methods include Root Mean Square of the Successive Difference of peak-to-peak intervals (RMSSD), Shannon entropy (ShE), Poincaré plot analysis (PPA), dynamic time warping for shape analysis, and spectral analysis^9–12^. These methods generally rely on distance-based metrics, which in many situations have been shown to yield unreliable results^5^.

Recently, convolutional neural networks (CNN), a class of artificial neural networks with a strong capability in feature extraction, have achieved great success in computer vision medical tasks^13–16^. Features are no longer hand-derived but learned by models trained through backpropagation^15^. CNN’s have become the dominant choice for many machine learning tasks due to their high discriminatory power in supervised machine learning settings, a facet that relies on large quantities of manually labeled data for building high-quality models. There are, however, limitations to CNNs including their sensitivity to weight initialization and their dependency on large-scale labeled training data. In some domains, like biomedical applications, it is very difficult to construct large-scale well-annotated datasets due to the cost of manual annotation, data acquisition, and patient privacy. This can limit development and accessibility. Thus, the ability to learn effectively from smaller datasets or unlabeled observations is critical to alleviating the bottlenecks in the advancement of this paradigm for improving healthcare.

Transfer learning aims to solve the problem of insufficient training data. In transfer learning the goal is to build models that adapt and generalize well, even when distributions between datasets used for training and testing differ. With the expansion of deep learning to medical applications, transfer learning has become integral to its success. Standard practice includes taking pretrained existing architectures commonly designed for natural images and fine-tuning it on a medical image dataset of interest. For 1-dimensional datasets, options for selecting pretrained architectures and fine-tuning that model on a dataset of interest are limited. As a result of this void in available pretrained 1-dimensional models, the ability to effectively train from smaller datasets or unlabeled observations becomes a critical task.

Researchers have applied deep CNN to the problem of AF event detection^8,17–21^. Shashikumar et al.^17^ developed a blended approach, combining the output of a CNN with other selected features derived from beat-to-beat variability and signal quality. This method required experts to define appropriate and critical features needed for success. Tison et al.^18^ proposed using averaged heart rates, step count, and time lapse as input for passive detection of AF using a neural network consisting of 8 layers, each of which had 128 hidden units. Last, Poh et al.^8^ proposed a method for dense CNN to distinguish between noise, sinus rhythm, ectopic rhythms, and AF across an ensemble of three simultaneously collected PPG signals. These AF classification methodologies do not consider joint estimations of signal quality assessment or explore transfer learning to boost discriminatory power. Providing a quality assessment score with each rhythm classification allows for high-quality scores to signify that a rhythm classification is more reliable. In addition, exploring transfer learning for AF detection appeals to biomedical research given the common challenge of limited access to large labeled cohorts.

To address this gap, we present DeepBeat, a method for the detection of AF from wrist-based PPG sensing. Our method combats the unique noise artifact problem common in AF detection by utilizing a multitask CNN architecture, transfer learning (TL), and an auxiliary signal quality estimation task for AF event detection from spatially segmented physiological PPG signals. Our main contributions are: [1] We propose that using a multitask learner for two correlated tasks, rhythm estimation and signal quality estimation, allows for collaborative learning and increased performance. [2] We evaluate the use of convolutional denoising autoencoders (CDAE) for unsupervised learning as a pretraining technique, part of a hybrid approach where pretrained weights are used in the foundational layers of DeepBeat. [3] Given the limited data often available in biomedical studies, we assess whether using simulated data as a pretraining technique leads to better distinction in learned class representations. [4] While examining our model in a prospective external validation study, we find that DeepBeat is robust to data acquired from different sources while maintaining high AF discriminatory power.

## Results

### Training the model

**Fig. 1:**
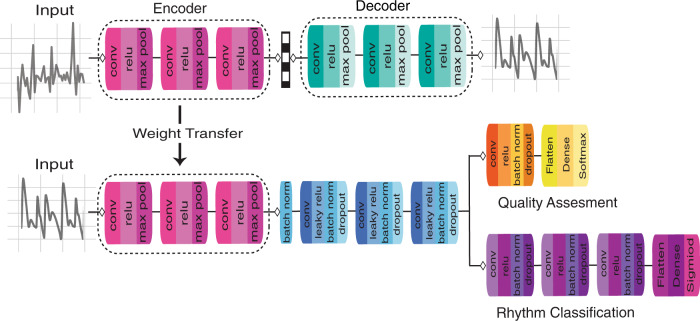
DeepBeat model architecture. The proposed model architecture for DeepBeat, two tasks are shown: (top) unsupervised pretraining and (bottom) supervised learning through fine-tuning. The top represents the pretraining process on the unlabeled simulated data, and the bottom represents the multitask fine-tuning process on the labeled data. The trained encoder weights serve as the foundational layers of the multitask model.

Training was broken into two phases: pretraining using CDAE on over one million simulated physiological signals and fine-tuning using transfer learning on a collected set of real-world data, Fig. 1. The real-world dataset is composed of data collected at Stanford University from participants undergoing elective cardioversions or elective stress tests and supplemented with a publicly available dataset from the IEEE Signal Processing Cup 2015 (Table 1). The data were split into training, validation, and test sets with no participants’ overlap between sets. The quantitative comparison of our models’ performance was evaluated on three datasets. The first is the held-out test set, the second is a publicly available pulse oximetry benchmark dataset, and the third is an external evaluation dataset collected from a different commercial device. The test dataset is reflective of the training set in terms of data acquisition in a controlled environment. The pulse oximetry dataset is currently the only PPG-based benchmark dataset publicly available. Although it measures respiratory rate and consists of non-AF monitoring from pulse oximetry, it can serve as an out-of-distribution estimation of false-positive rates. The external evaluation dataset represents real-world use, where the signals are recorded over longer periods of time and may include a higher degree of artifact due to uncontrollable environmental variation. We provide a comparison of all datasets in Supplementary Tables 1 and 2.

**Table 1:** Demographics of study participants collected for DeepBeat development.

	Cardioversion cohort	Exercise stress test cohort	Ambulatory cohort
Number of subjects	107	41	15
Number of subjects with atrial fibrillation	107	0	4
Mean age	68	56	67
Sex (M/F)	85/22	26/14	11/4

### Performance evaluation criteria

**Fig. 2:**
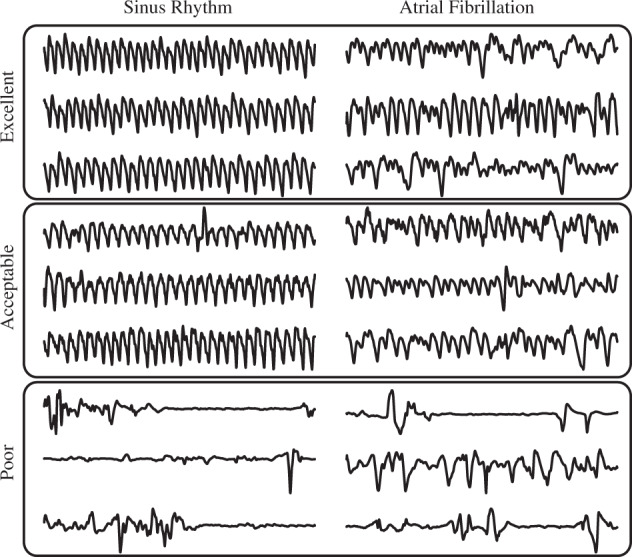
Example of rhythm and quality assessments of training data. Examples of physiological signals grouped by assessment scores used to train and evaluate the DeepBeat model. From top to bottom, quality assessment scores of excellent, acceptable, and poor. Left column are signals from participants in normal sinus rhythm and the right column are signals from participants in atrial fibrillation.

DeepBeat takes pre-specified windowed physiological signal data of time length *t* (25 s) as input and performs two prediction tasks, signal quality assessment, and AF rhythm classification. Figure 2 provides examples of physiological signals and quality assessment scores that were used to train and evaluate the method. We systematically compare the performance of the model on both a held-out test data, publicly available dataset and the external evaluation dataset. Table 2 reports the test performance of all models explored. All evaluation metrics were calculated using the weighted macro-averaged of the following: sensitivity/recall (the fraction of the expert diagnoses that are successfully retrieved), specificity (the fraction of predicted negatives that match the true negatives), false-negative rate (the conditional probability of a positive result given an event that was not present), false-positive rate (the conditional probability of a negative result given that the event is present), and F1 (the harmonic mean of precision and recall). While accuracy is classically used for evaluating overall performance, we chose not to consider it here due to class imbalance, F1 score is a more appropriate metric for ascertaining detection rates for rare AF detection.

**Table 2:** Performance of classification models.

Model	Sensitivity	Specificity	False positive rate	False-negative rate	F1 score
Random forest	0.32	0.79	0.21	0.68	0.39
VGG16: single task (AF)	0.92	0.71	0.29	0.08	0.64
DeepBeat: single task (AF)	0.49	0.90	0.10	0.51	0.54
DeepBeat: single task with pretraining (AF)	0.52	0.88	0.12	0.48	0.56
DeepBeat: multitask (AF + QA)	0.97	0.89	0.11	0.03	0.71
DeepBeat: multitask with pretraining (AF + QA)	0.98	0.99	0.01	0.02	0.96

### Multitask learning is essential for high classification accuracy of AF observations

Training a single model on multiple tasks with shared encoding can improve a model’s performance on all tasks, as different tasks serve as implicit regularization to prevent the model from overfitting to a particular task^22^. We conducted experiments to study the effect of a single-task learner (STL) against multitask learner (MTL). For a STL, only AF detection is predicted per input window, in the case of MTLs, AF detection and signal quality assessment are both predicted per window. Table 2 shows the model performances on the held-out test set of a STL versus MTL. First, we see that MTL has a profound effect on the performance of sensitivity rates, which increase from 0.49 to 0.97. Next, we see a decrease in false-negative rates from 0.51 to 0.3, the additional QA auxiliary task in the MTL method permits signal QA thresholding to occur consequently removing false-negative signals attributed to poor signal quality.

### Pretraining using CDAE increases classification accuracy

In order to evaluate the effects of using CDAE as a pretraining method and examine its impact on performance, we systematically compare the DeepBeat architecture with CDAE pretraining and without. For both model versions, pretrained with CDAE and random initialization, training was conducted under similar conditions and under the same parameters. The results in Table 2 suggest that using the extracted encoder from the trained CDAE as a form of unsupervised pretraining results in substantially higher performance across all metrics. The false-positive rates dramatically decrease from 0.11 to 0.01 and specificity increases from 0.89 to 0.99. These results show that the proposed multitask DeepBeat, pretrained with CDAE achieves high performance and using only a subset of these components, only single-task learning or only pretraining, leads to poorer test performance. For the sake of completeness, we also consider a random forest method and an adapted 1D version of the standard architecture for natural images, the VGG12 architecture, for a baseline comparison against Deepbeat. The details of the two additional models are provided in the Supplementary Notes 1 and 2. Results from those methods are also highlighted in Table 2 and each performs significantly less well than DeepBeat.

### Interpreting model predictions

In order to improve our understanding of how DeepBeat classifies AF events, we implemented a simple class activation map for visualization. Heatmaps of class activations over input signals were used to visualize how each data point within the signal influences the model’s predictions.

**Fig. 3:**
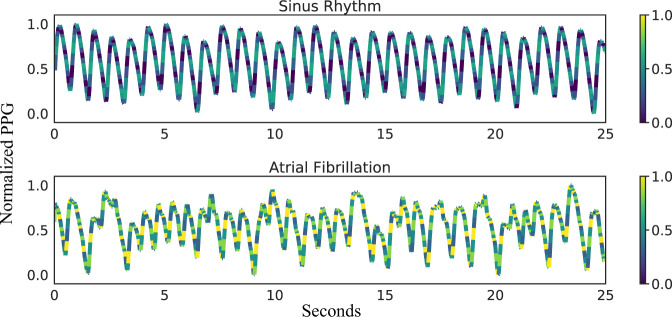
Differences in class activation map by rhythm classification. Example of signals from held-out test dataset. The predicted class score is mapped back to the last convolutional layer to generate the class activation maps (CAMs). The CAM highlights the class-specific discriminative regions between sinus (top) and atrial fibrillation (bottom).

**Fig. 4:**
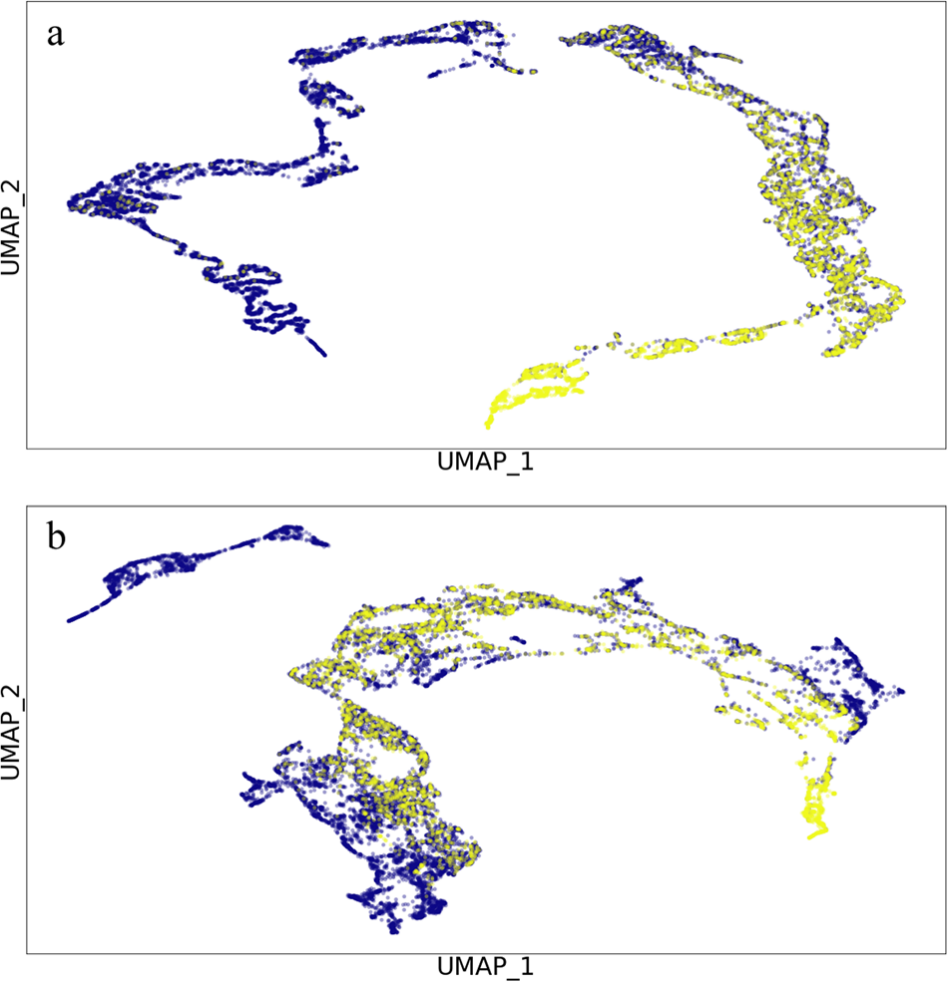
Visualization of learned rhythm class distinction. UMAP representation from the last (Dense_18) layer of the DeepBeat model. The two colors represent the two classes, respectively, normal sinus rhythm as purple, and yellow as atrial fibrillation. Top panel (**a**): pretrained network, bottom (**b**), random initialization. The distinction between the two classes is clear in the pretrained network compared to random initialization.

We emphasize a set of AF and non-AF signals randomly chosen from the test partition to highlight saliency scores; the higher the saliency score, the lighter the color and the more influential the region is to the model’s prediction, Fig. 3. From the figure, we identify regions of the signal that are most discriminative for classification. For example, there are clear global differences between the AF and non-AF signals, with stark differences in the upslope of PPG signals across the classes. Thus, we can infer the regions of the upslope from the systolic phase to be informative for AF class-specific predictions. In addition, we conducted additional experiments to study the effect of transfer learning for the learned representations of DeepBeat. We extract the output of the last dense layer before rhythm prediction is made and use UMAP^23^ to visualize the learned distinction of non-AF versus AF event windows in Fig. 4. As a baseline comparison we also consider the learned representations from the extracted output when training was performed from random initialization. In the pretrained model visualisation, there is a clear distinction between non-AF and AF event windows, whereas the contrast between representations from the random initialization is not clearly apparent.

### DeepBeat achieves strong performance on external data

Achieving high discriminatory power on a test dataset originating from the same population distribution as the training dataset is critical. Transferring that same trained model to a different population is a greater and much more relevant test with respect to translation to practice. In order to determine the robustness of DeepBeat, we evaluated the performance on two different external datasets: the first is a freely available pulse oximetry benchmark dataset, and the second is a prospective study of 15 free-living individuals. The pulse oximetry dataset, is currently the only publicly available PPG-based dataset, and consists of non-AF monitoring. It will be used as an out-of-distribution estimate of false-positive rates. The second dataset was collected for this study and comprised ambulatory PPG monitoring recorded over the course of 1 week using an independently engineered wrist-worn device while participants simultaneously wore an ECG rhythm patch^24^. Rhythm was determined by two cardiologists’ manual annotations following computerized reference ECG algorithms. In the case that the two clinicians disagreed, a third senior cardiologist was asked to review annotations. For both datasets, the data from each individual was partitioned into 25-s non-overlapping windows. Metrics were calculated for selected windows meeting the excellent signal quality in Table 3. In the pulse oximetry benchmark, due to the composition of only individuals with non-AF events, we only consider specificity and false-positive rates. For both specificity and false-positive rate, DeepBeat correctly classified all signals as non-AF events, with no false-positive cases. For the ambulatory cohort, 11 of the 15 monitored individuals had no confirmed AF episodes during their 1-week period. These individuals were used to estimate the false positive rates this algorithm would have in an everyday environment for high-risk populations (Table 3). Our results show less than a 0.007 false-positive detection rate across these individuals. Four individuals had a confirmed AF event during the 2 weeks, resulting in a total of 929 25-s AF annotated episodes. DeepBeat classified AF presence in all 4/4 individuals (Table 3), detecting 925 episodes correctly for a combined sensitivity rate of 0.98. The results, in Table 3, suggest that DeepBeat’s performance in accurately predicting AF event episodes was robust to different populations, wearable devices, and hospital vs ambulatory environments.

**Table 3:** External evaluation of DeepBeat.

Dataset	Sensitivity	Specificity	False-positive rate	False-negative rate	F1 score
Ambulatory monitoring (*n* = 15)	0.98	0.99	0.007	0.01	0.93
Capnobase, pulse oximetry (*n* = 42)	n/a	1	0	n/a	n/a

## Discussion

The primary contributions of this work are as follows:

1. We introduce a multitask CNN method, DeepBeat, to model the intrinsic properties of physiological PPG signals. The proposed algorithm performs collaborative multitask feature learning for two correlated tasks, input signal quality assessment and event detection (AF presence).
2. The model benefits from unsupervised transfer learning from pretraining using convolutional denoising autoencoders (CDAE) on simulated physiological signals. The ability of CDAEs to extract repeating patterns in the input makes them suitable to be used to extract true physiological signals from noise-induced inputs.
3. We visualize the differences of transfer learning versus random initialization on the projected representations learned by Deepbeat.
4. Last, we explore the robustness of DeepBeat in two external studies: a publicly available pulse-oximeter benchmark dataset and a prospective study in free-living ambulatory individuals monitored over a 1-week time span. We find that DeepBeat maintains high discriminatory power in both cohorts.

Reproducibility is a critical aspect of any clinical physiological measurement. In this work, we show the utility of scoring and incorporating signal quality assessment for event detection in a jointly trained deep learning approach. Incorporating a quality score allows for filtering of unusable signals and assists in achieving high-performance metrics across sensitivity, specificity, and F1 scores in a 25 s sampling window. The performance improvements could be attributed to two complementary components of the method: (1) unsupervised transfer learning through pretraining using a CDAE on a simulated dataset, and (2) the use of a multitask learning architecture, which had different capabilities in generalizing and adapting to different training objectives.

CDAEs have been applied for unsupervised pretraining^22^ and can be categorized as data-driven network initialization methods or a special type of semi-supervised learning approach^25^. To the best of our knowledge, our study is the first to incorporate simulated data in conjunction with transfer learning for AF detection in wearable devices. With the rise of deep learning in medical imaging applications, transfer learning has become common. Large models pretrained on natural image datasets, such as ImageNet, are fine-tuned on a medical image database of choice. Large pretrained image models cannot be easily imported to fine-tune on 1-dimensional data, leaving few options for non-imaging data. We show that an adapted 1D VGG architecture had significantly lower performance. Non-standard, smaller and simpler convolutional networks have also been shown to perform comparably to standard ImageNet models for medical datasets despite having significantly worse accuracy on ImageNet^26^. Raghu et al. argue the indication of strong performance metrics on natural images is not necessarily a good indication of success on limited medical datasets^26^, a result confirmed with our analysis. Given the considerable differences in 1D and 2D data, using standard image-based networks may not be an appropriate choice. Furthermore, large computationally expensive models might be infeasible on mobile or wearable applications, furthering the need for simple, lightweight models.

Following the success of other multitask learning neural networks^27^, DeepBeat’s architecture for AF event detection and signal quality assessment was designed to leverage several advantages that multitask learning neural networks offer. The DeepBeat architecture utilizes low-level feature sharing by allowing the signal quality assessment task and AF event-detection task to collectively learn the same feature representation through the use of shared layers or hard parameter sharing. This is motivated by the following: First, learned features for the signal quality assessment task provide value for analyzing regions for which AF events are present or absent. Second, shared layers encourage a reduction in the number of parameters needed for the network to generalize to a greater range of individuals. The integration of signal context information is beneficial. Difficulties arise when distinguishing between AF and non-AF events when signals are corrupted by noise or artifact. One approach to overcoming these obstacles involves the provision of context information on signal quality so the learned features are tandem with features predictive of AF events. The event-detection task determines whether a window contains regions of AF events, while the signal assessment task predicts the quality of the signal. Preservation of important physiological signal information throughout the model should not be translation invariant, i.e., the learned features from the signal assessment task should be preserved in the AF detection outputs.

Previously, Poh et al.^8^ reported a method using dense convolutional neural networks (DCNN) with lower sensitivity (recall) of 95.2%, and PPV (precision) of 72.7% using single-channel PPG as input to differentiate between AF and non-AF signals. This study included a large number of participants, but a very different methodological design. The authors consider noise as an equally likely category as AF and non-AF. The DCNN approach may limit the ability to disentangle the impact that signal quality has on predicted AF versus non-AF states. In addition, the DCNN architecture consisted of six dense blocks resulting in a model with a total of 201 layers, significantly deeper than the model we propose. Employing transfer learning with a much shallower network as seen with DeepBeat can increase the precision of AF events detected and an over-parameterized model may not be necessary.

A substudy of the eHeart study evaluated the applicability of AF detection using a smartwatch^18^. The study design was broken into three distinct phases in which the accuracy to detect AF was moderate in the ambulatory stage. The sensitivity (0.98) for the validation phase was comparable to the sensitivity of DeepBeat, but DeepBeat outperforms on specificity. The reduced specificity of that model could be contributed to by the input data, use of averaged heart rates, step count, and time lapse. The ability to correctly identify average heart rate is critical for high-performance success and conditions such as high-intensity motion or improper wear can greatly impact heart rate calculations. Our method is not based on calculated heart rate but instead infers rhythm directly from the raw waveform, reducing the need to calculate heart rate and reducing possible error propagation throughout the model.

The Apple Heart Study was focused on ambulatory AF detection with a proprietary algorithm based on irregular tachograms (periodic measurements of heart rate regularity). The study enrolled 419,297 individuals and monitored for a median of 8 months. Among participants who were notified of an irregular pulse, the positive predictive value was 0.84 (95% CI, 0.76–0.92) for observing AF on the ECG simultaneously with a subsequent irregular pulse notification and 0.71 (97.5% CI, 0.69–0.74) for observing AF on the ECG simultaneously with a subsequent irregular tachogram^28^. While not directly comparable (overall sensitivity is not estimable in the Apple Heart Study due to study design), the performance of Deepbeat reported here would suggest favorable performance when deployed at scale.

Our study has limitations. We only considered one abnormal cardiac rhythm (albeit the most common one). The training data derived were a mixture of healthy individuals and patients who were hospitalized long term or for same day outpatient procedures (i.e., taken from a population with a much higher prevalence of arrhythmia than the general population). When comparing reported evaluation metrics, our algorithm outperforms other deep learning methods that have been proposed so far for AF event detection^8,17–21^. However, a direct comparison of prior published work is challenging due to the lack of published code or availability of trained models and training data needed for baseline neural network-based comparisons.

In summary, event detection of AF through the use of wearable devices is possible with strong diagnostic performance metrics. We achieve this through signal quality integration, data simulation and the use of CDAEs which are highly suitable for deriving features from noisy data. In light of the increased adoption of wearable devices and the need for cost-effective out-of-clinic patient monitoring, our method serves as a foundational step toward future studies involving extended rhythm monitoring in high-risk AF individuals or large-scale population AF screening. Further studies will be needed to determine whether the benefits of such diagnostic screening result in improved patient outcomes.

## Methods

### Human studies

The source data used for training DeepBeat comprised a combination of a novel data generated for this study and publicly available data. Pretraining using CDAE was trained with a novel PPG simulated dataset, and DeepBeat was developed using participants from three datasets, two from Stanford hospital, first participants undergoing elective cardioversions and secondly, participants performing elective stress tests. The third dataset is a publicly accessible 2015 IEEE Signal Processing Cup Dataset was used to supplement the Stanford dataset to provide out-of-institution examples. For an additional evaluation, a pulse-oximeter benchmark data and a study from an ambulatory cohort were used to evaluate algorithm performance. The participant demographic summary can be found in Table 1. All studies conducted at Stanford were conducted in accordance with the principles outlined in the Declaration of Helsinki and approved by the Institutional Review Board of Stanford University (protocol ID 35465, Euan Ashley). All participants provided informed consent prior to the initiation of the study.

The simulation of synthetic physiological signals was generated and built upon RRest, a simulation framework^29^. The simulation framework for synthetic physiological signals was expanded to include a combination of baseline wander and amplitude modulation for simulation of sinus rhythm physiological signals. For simulations of an AF state, a combination of frequency modulation, baseline wander, and amplitude modulation was simulated. Frequency modulation was applied to specifically to mimic the chaotic irregularity of an AF rhythm. This assumption was the foundation for all simulations of AF signals. In addition to the expanded simulation version, an additional noise component was added to the simulated signals based on a Gaussian noise distribution. This provides the capability to simulate high-quality signals in the presence of low noise and low-quality signals in the presence of high noise. We simulated sinus rhythm and AF states under different levels of Gaussian noise to best represent observed real-world scenarios, further details can be found in Supplementary Fig. 1.

The collection of physiological signals before cardioversion were extracted from a wrist-based PPG wearable device worn by participants at Stanford hospital undergoing direct current cardioversion for the treatment of AF. The study included participants with an AF diagnosis who were scheduled for elective cardioversion. We included all adult participants able to provide informed consent and willing to wear the device before and after the CV procedure. We included all participants with an implanted pacemaker or defibrillator and who also had planned or unplanned transesophageal echocardiogram. In total, 132 participants were recruited and monitored; data from 107 were of sufficient duration and quality to be included in this study. The average monitoring time was ~20 min post and 20 min prior to the CV. All physiological signals were sampled at 128 Hz and wirelessly transmitted via Wifi to a cloud-based storage system.

The collection of physiological signals from exercise stress test were extracted from a wrist-based PPG wearable device worn by participants at Stanford hospital who were scheduled for an elective exercise stress test. We included all adult participants who were able to provide informed consent and willing to wear the device during an elective exercise stress test. In total, 42 participants were monitored; data from all 42 participants were included in this study. The average monitoring time was ~45 min. All physiological signals were sampled at 128 Hz and wirelessly transmitted via Wifi to a cloud-based storage system.

The PPG database from the 2015 IEEE Signal Processing Cup^30^ was included in this study to provide a source of data from healthy non-AF participants. The dataset consists of two channels of PPG signals, three channels of simultaneous acceleration signals, and one channel of simultaneous ECG signal. PPG signals were recorded from a participant’s wrist using PPG sensors built-in a wristband. The acceleration signals were recorded using a tri-axial accelerometer built into the wristband. The ECG signals were recorded using standard ECG sensors located on the chest of participants. All signals were sampled at 125 Hz and wirelessly transmitted via Bluetooth to a local computer.

The pulse-oximeter benchmark dataset was downloaded from the on-line database CapnoBase.org. The dataset consists of individuals randomly selected from a larger collection of physiological signals collected during elective surgery and routine anesthesia for the purpose of development of improved monitoring algorithms in adults and children^31^. The PPG signals were recorded at 100 Hz with S/5 Collect software (Datex-Ohmeda, Finland)^31^.

A prospective cohort of 15 participants with paroxysmal AF were recruited prospectively for a free-living ambulatory monitoring for an average of 1 week. Participants wore a wrist-based PPG wearable device together with an ECG reference device. During the monitoring period, participants were asked to continue with their regular daily activities in their normal environment. PPG signals were extracted from the device after study was complete and clinically-annotated ECG rhythm annotations were provided from the reference ECG device.

### Data preprocessing

Preprocessing of the simulated physiological signals for CDAE consisted of partitioning the data into training, validation, and test partitions. Simulated physiological PPG signals consisted of 25 s time frames. The collected physiological signals were partitioned into training, validation, and test partitions with no individual overlap between each set. We used overlapping windows for the training set as a data augmentation technique to increase the number of training examples. All signals were standardized to [0, 1] bounds and bandpass filtered and downsampled by a factor of 4. Supplementary Table 1 illustrates the number of signals for each partition from the Stanford cardioversion, exercise stress test, and IEEE signal challenge datasets.

### Signal quality assessment

To train a multitask model assessing both signal quality and event detection, signal quality labels were needed for each signal window. Event-detection labels were known, given the datasets and timestamp the signal originated from. To provide a signal quality assessment label for the training set we created an expert scored dataset of PPG signals known as the signal quality assessment dataset. For each time window set considered, 1000 randomly selected windows were scored and partitioned into a train, validate and test sets. Each window was scored according to 1 of 3 categories (excellent, acceptable, and noise) in the concordance of published recommendations for PPG signal quality^7^. The signal quality classes were based on published standardized criteria (Elgendi quality assessments^7^). A separate model for QA was trained using the scored dataset as outcomes and used to predict quality labels for the remaining unscored windows considered.

### Algorithm

Pretraining was performed using unsupervised pretraining using convolutional denoising autoencoders. Autoencoders are a type of neural network that is composed of two parts, an encoder, and decoder. Given a set of unlabeled training inputs, the encoder is trained to learn a compressed approximation for the identity function so that the decoder can produce output similar to that of the input, using backpropagation. Consider an input $x \in \Re ^d$ being mapped to a hidden compressed representation $y \in \Re ^d$ by the encoder function: *Encoder*: $y = h_\theta (x) = \sigma (Wx + b),$ where *W* is the weights matrix, *b* is the bias array, *θ* = {*W*, *b*}, and *σ* can be any nonlinear function such as ReLu. The latent representation *y* is then mapped back into a reconstruction *z*, with the same shape as input *x* using a similar mapping: *Decoder*: $z = \sigma (W^\prime y + b^\prime )$. The reconstruction of the autoencoder attempts to learn the function such that $h_\theta (x) \approx x$, to minimize the mean-squared difference $L(x,z) = {\sum} {(x - h_\theta (x))^2}$. Convolutional denoising autoencoders (CDAE) are a stochastic extension to traditional autoencoders explained above. In CDAE, the initial input *x* is corrupted to $\underline x$ by a stochastic mapping $\underline x = C(\underline x |x)$, where *C* is a noise generating function, which partially destroys the input data. The hidden representation *y* of the *k*th feature map is represented by $y^k = \sigma (W^k \ast x + b)$, where * denotes the 1D convolutional operation and *σ* is a nonlinear function. The decoder is denoted by $z \approx h_\theta (x) = \sigma (\mathop {\sum}\nolimits_{i \in m} {m^i} \ast \underline W + b)$, where *m* indicates the group of latent feature maps and $\underline W$ is the flipped operation over the dimensions of *W*^32^. Compared to traditional autoencoders, convolutional autoencoders can utilize the full capability of CNN to exploit structure within the input with weights shared among all input locations to help preserve local spatiality^32^.

We simulated a training dataset for artifact induced PPG signals and its corresponding clean/target signal. We use convolutional and pooling layers in the encoder, and upsampling and convolutional layers in the decoder. To obtain the optimal weights for *W*, weights were randomly initiated according to He distribution^33^ and the gradient calculated by using the chain rule to back-propagate error derivatives through the decoder network and then the encoder network. Using a number of hidden units lower than the inputs forces the autoencoder to learn a compressed approximation. The loss function employed in pretraining was mean-squared error (MSE) and was optimized using a back-propagation algorithm. The input to the CDAE was the simulated signal dataset with a Gaussian noise factor of 0.001, 0.5, 0.25, 0.75, 1, 2, and 5 added to corrupt the simulated signals. The uncorrupted simulated signals are then used as the target for reconstruction. We used three convolution layers and three pooling layers for the encoder segment and three convolution layers and three upsampling layers for the decoder segment of the CDAE, Supplementary Table 3. ReLU was applied as the activation function and Adam^34^ is used as the optimization method. Each model was trained with MSE loss for 200 epochs with a reduction in learning rate by 0.001 for every 25 epochs if validation loss did not improve. Further results from the CDAE training can be found in the Supplementary Note 3, Supplementary Fig. 2, and Supplementary Table 4.

Transfer learning is an appealing approach for problems where labeled data is acutely scarce^35^. In general terms, transfer learning refers to the process of first training a base network on a source dataset and task before transferring the learned features (the network’s weights) to a second network which is trained on an external and sometimes related dataset and task. The power of transfer learning is rooted in its ability to deal with domain mismatch. Fine-tuning pretrained weights on the new dataset is implemented by continuing backpropagation. It has been shown that transfer learning reduces the training time by reducing the number of epochs needed for the network to converge on the training set^36^. We utilize transfer learning here by extracting the encoder weights from the pretrained CDAE and copy the weights to the first three layers of the *DeepBeat* model architecture. A similar approach has been applied before successfully in related ECG arrhythmia detection^22^. The motivation behind using CDAE for unsupervised pretraining on simulated physiological signals was to provide the earlier foundational layers of the *DeepBeat* model the ability to quickly identify learned features that constitute important physiological signal elements.

DeepBeat was trained to classify two tasks through a shared structure. The input is a single physiological PPG signal. The convolutional layers of the first three layers include receptive field maps with initialized filter weights from the pretrained CDAE encoder section. Three additional layers were added after the encoder section, leading to a total of six shared hidden layers. For hidden layers 4–6, leaky rectified linear unit^37^, batch normalization^38^, and dropout layers and convolutional parameters were selected through hyperparameter search. Model specification can be found in Supplementary Table 5.

The quality assessment task (QA) and AF event-detection task builds upon the six shared layers branching into two specialized arms, the QA task and event-detection task. The QA arm consists of an additional convolutional layer, rectified linear unit (ReLU), batch normalization, dropout and two dense layers before final softmax activation for classification is used. The event-detection task consisted of three additional convolutional layers, each followed by a ReLU, batch normalization, and dropout layers. Two additional dense layers were added before final softmax activation. For all layers except the pretrained encoder, weights were randomly initiated according to He distribution^33^. With a given training input, predictions for both tasks, QA and rhythm event detection were estimated and backpropagation was used to update the weights and the corresponding gradients throughout the network. Hyperparameter optimization for the number of layers, activation functions, receptive field map size, convolutional filter length, epochs, and stride was performed using hyperas^39^. The best performing model was selected by highest F1 score on the validation data. We implemented DeepBeat using Python 3.5 and Keras^40^ with Tensorflow 1.2^41^. We trained the model on a cluster of 4 NVIDIA P100 GPU nodes.

### DeepBeat performance metrics

Classification performance metrics were measured in two ways, per episode and weighted macro-averaged across individuals using the following metrics: sensitivity, specificity, false-positive rate, false-negative rate, and F1 score. While accuracy is classically used for evaluating overall performance, F1 scores are more useful with significant class imbalance, as is the case here. Weighted macro-averaged are reported within the tables.

### Reporting summary

Further information on research design is available in the Nature Research Reporting Summary linked to this article.

## Supplementary information

Supplemental Information

Reporting Summary

## Data Availability

The data used to train DeepBeat (train, validate and test partitions) is available through synapse (Synapse ID: syn21985690). Ambulatory dataset was made available to Stanford for the current study and is not publicly available.

## Code availability

Trained DeepBeat model and code details can be found at https://github.com/AshleyLab/deepbeat.
