# Supplementary material for: Multi-task deep learning for cardiac rhythm detection in wearable devices
Source: NPJ Digit Med. 2020 Sep 9;3:116. doi: 10.1038/s41746-020-00320-4 (PMC7481177; doi:10.1038/s41746-020-00320-4)
Supplement: Supplementary file 1 — Supplemental Information [file 41746_2020_320_MOESM1_ESM.pdf]

Supplementary Note 1

A feature-based approach has low discriminatory power for the detection of AF

For a baseline comparison, we investigate the effectiveness of a traditional feature-based machine learning method against our model. Baseline results are reported in Table 1. Nine features most commonly used in feature-based AF detection and signal quality estimation algorithms[9–11] were calculated for input into a MultiOutputClassifier random forest model from scikit-learn[40]. DeepBeat substantially outperforms the trained random forest model for AF detection across all metrics considered. The random forest results were dramatically less effective for detecting AF events compared to DeepBeat's AF event metrics. Our model's notable improvement in AF detection over other feature-based methods is reflective across all DeepBeat versions examined, demonstrating that a feature-based approach fails to have high discriminatory power for AF detection.

Details regarding random forest

To investigate the choice of a multi-task model, a comparison of different methods was performed. For a feature-based approach, random forests were used due to its capability of finding complex nonlinear relationships in data. The following features were calculated: kurtosis, skew, entropy, zero crossings, hjorthe mobility, hjorthe complexity, normalized root mean of successive differences, and Shannon entropy. A MultiOutputClassifier random forest model with n_estimators=100 and random_state=1 was used as parameters for training.

Supplementary Note 2

Details regarding 1D VGG

To investigate the choice of deep learning models, we selected the popular VGG12 architecture and adapted it for 1D input. Details of specifics regarding model architecture can be found in Table S6. Training of 1D VGG was similar to the approach used for DeepBeat, weights were randomly initiated according to He distribution [31] and hyperas was used for optimal selection of epoch, batch size and learning rates.

Supplementary Note 3

Results from unsupervised pre-training using convolutional *denoising autoencoders*

Results from the trained CDAE on scored signal quality assessment dataset can be found in supplemental Table S2**.** The mean squared errors were 0.0095, 0.0104, 0.0.0143 for excellent, acceptable and poor categories for the 25 second time segments. We found the lowest mean squared error for signal reconstruction in the excellent category across all time segments, suggesting that the trained CDAE is selecting filters appropriate for high-quality physiological signal reconstruction.

In order to determine that the CDAE was not introducing modulations typical of physiological signals when there was no physiological signal present, we performed a sensitivity analysis. Five hundred random signals were generated and ran through the trained CDAE model. The estimated MSE of the randomly generated noise was similar to that of the estimated MSE for the poor signal quality category across all time points. To further explore the estimated reconstruction predictions from the output of the trained CDAE, predictions were compared to

3rd order Savitzky-Golay filters. Mean squared error of the reconstruction CDAE prediction of the randomly generated noise set was 0.026, and the mean squared error of the 3rd order Savitzky-Golay filters was 0.023. These results confirm that the trained CDAE model provides a set of filters sensitive to frequencies unique to physiological signals and, in situations where no viable physiological signal is present, CDAE instead acts as a smoothing filter (Supplementary Figure 2).

Supplementary Figure 1

Simulated signals from dataset A. Left column, simulated sinus rhythm, top to bottom in increasing order of added Gaussian noise mixture (0.001, 0.15, 0.5, 0.75, 1, 2, 5). Right column, simulated AF rhythm, top to bottom in increasing Gaussian noise mixture (0.001, 0.15, 0.5, 0.75, 1, 2, 5). Pictured below all signals were simulated at 60 beats per minute (BPM).

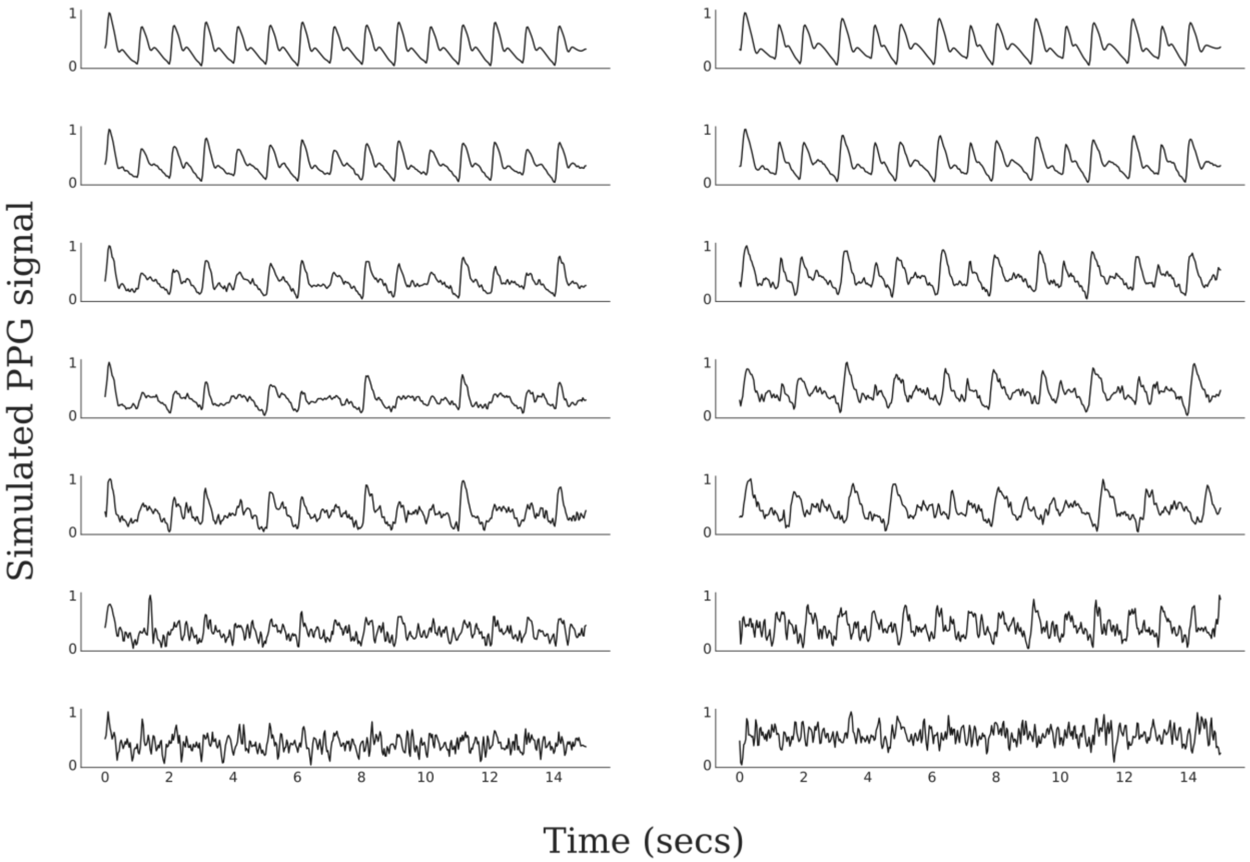

Supplementary Figure 2

Results from trained CDAE on denoising simulated signals, collected signal and random noise.

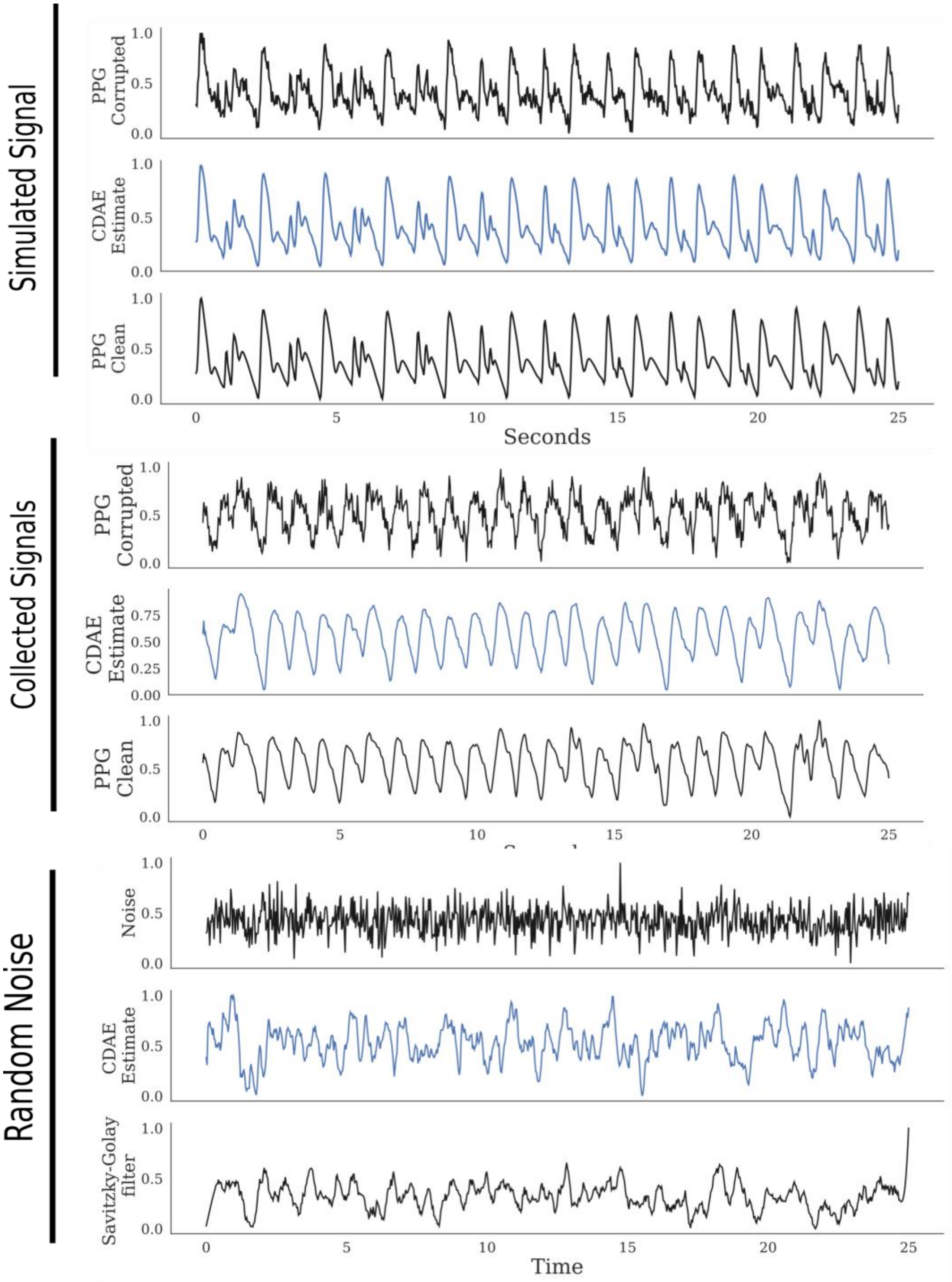

Supplementary Table 1

Breakdown of data samples by rhythm and quality assessment

partition	rhythm	QA	Count
**train**	sinus	poor	999,253
		acceptable	140,020
		excellent	390,851
	AF	poor	972,955
		acceptable	141,004
		excellent	159,851
**validate**	sinus	poor	295,449
		acceptable	56,572
		excellent	119,154
	AF	poor	33,691
		acceptable	8,075
		excellent	5,841
**test**	sinus	poor	8,856
		acceptable	1,718
		excellent	2,813
	AF	poor	3,483
		acceptable	314
		excellent	433

Supplementary Table 2

Comparison of evaluation data sets

	Number of patients	Total number of windows	Total number of AF windows
**Held out test set**	22	17,617	4,230
**Ambulatory cohort**	15	20,492	2,048

Supplementary Table 3

CDAE model architecture specifications

Layer Type	Output Shape	Param #
**Encoder**		
InputLayer	(None, 800, 1)	0
Conv1D	(None, 800, 64)	704
MaxPooling	(None, 266, 64)	0
Conv1D	(None, 266, 45)	23,085
MaxPooling	(None, 88, 45)	0
Conv1D	(None, 88, 50)	11,300
MaxPooling	(None, 44, 50)	0
**Decoder**		
Conv1D	(None, 44, 50)	12,550
UpSampling	(None, 88, 50)	0
Conv1D	(None, 88, 45)	18,045
UpSampling	(None, 264, 45)	0
Conv1D	(None, 264, 64)	28,864
UpSampling	(None, 792, 64)	0
Flatten	(None, 50688)	0
Dense	(None, 800)	40,551,200

Supplementary Table 4

CDAE mean squared error for signal reconstruction

	Excellent	Acceptable	Poor
**25 seconds**	0.0095	0.0104	0.0143

Supplementary Table 5

DeepBeat model architecture specifications

Layer Type	Output Shape	Param #
**Extracted Encoder**		
InputLayer	(None, 800, 1)	0
Conv1D	(None, 800, 64)	704
MaxPooling	(None, 266, 64)	0
Conv1D	(None, 266, 45)	23,085
MaxPooling	(None, 88, 45)	0
Conv1D	(None, 88, 50)	11,300
MaxPooling	(None, 44, 50)	0
**Shared layers**		
BatchNormalization	(None, 44, 50)	200
Conv1D	(None, 15, 64)	12864
Leaky ReLu	(None, 15, 64)	0
BatchNormalization	(None, 15, 64)	256
Dropout	(None, 15, 64)	0
Conv1D	(None, 5, 35)	8995
Leaky ReLu	(None, 5, 35)	0
BatchNormalization	(None, 5, 35)	140
Dropout	(None, 5, 35)	0
Conv1D	(None, 5, 64)	9024
Leaky ReLu	(None, 5, 64)	0
BatchNormalization	(None, 5, 64)	256
Dropout	(None, 5, 64)	0

**Rhythm Branch**		
Conv1D	(None, 2, 35)	11235
BatchNormalization	(None, 2, 35)	140
Dropout	(None, 2, 35)	0
Conv1D	(None, 1, 25)	525
BatchNormalization	(None, 1, 25)	100
Dropout	(None, 1, 25)	
Conv1D	(None, 1, 35)	2660
BatchNormalization	(None, 1, 35)	140
Dropout	(None, 1, 35)	0
Flatten	(None, 35)	0
Dense	(None, 175)	6300
Dense	(None, 2)	352

**Quality Assessment Branch**		
Conv1D	(None, 3, 25)	6425
BatchNormalization	(None, 3, 25)	100
Dropout	(None, 3, 25)	0
Flatten	(None, 75)	0
Dense	(None, 175)	13300
Dense	(None, 3)	528
