# Supplementary material for: Multi-task deep learning for cardiac rhythm detection in wearable devices
Source: NPJ Digit Med. 2020 Sep 9;3:116. doi: 10.1038/s41746-020-00320-4 (PMC7481177; doi:10.1038/s41746-020-00320-4)
Supplement: Supplementary file 2 — Reporting Summary [file 41746_2020_320_MOESM2_ESM.pdf]

nature research

Corresponding author(s): Euan Ashley

Last updated by author(s): Jul 13, 2020

Reporting Summary

Nature Research wishes to improve the reproducibility of the work that we publish. This form provides structure for consistency and transparency in reporting. For further information on Nature Research policies, see our Editorial Policies and the Editorial Policy Checklist.

Statistics

For all statistical analyses, confirm that the following items are present in the figure legend, table legend, main text, or Methods section.

n/a	Confirmed	
☐	☒	The exact sample size (*n*) for each experimental group/condition, given as a discrete number and unit of measurement
☐	☒	A statement on whether measurements were taken from distinct samples or whether the same sample was measured repeatedly
☒	☐	The statistical test(s) used AND whether they are one- or two-sided<br>*Only common tests should be described solely by name; describe more complex techniques in the Methods section.*
☒	☐	A description of all covariates tested
☒	☐	A description of any assumptions or corrections, such as tests of normality and adjustment for multiple comparisons
☒	☐	A full description of the statistical parameters including central tendency (e.g. means) or other basic estimates (e.g. regression coefficient) AND variation (e.g. standard deviation) or associated estimates of uncertainty (e.g. confidence intervals)
☒	☐	For null hypothesis testing, the test statistic (e.g. *F*, *t*, *r*) with confidence intervals, effect sizes, degrees of freedom and *P* value noted<br>*Give P values as exact values whenever suitable.*
☒	☐	For Bayesian analysis, information on the choice of priors and Markov chain Monte Carlo settings
☐	☒	For hierarchical and complex designs, identification of the appropriate level for tests and full reporting of outcomes
☒	☐	Estimates of effect sizes (e.g. Cohen's *d*, Pearson's *r*), indicating how they were calculated

Our web collection on statistics for biologists contains articles on many of the points above.

Software and code

Policy information about availability of computer code

Data collection	https://developer.artik.cloud/ was used to extracting data from wearables to a cloud based system.
Data analysis	scikit-learn 0.21.3 , Python 3.5 and Keras with Tensorflow 1.2. We trained the model on a cluster of 4 NVIDIA P100 GPU nodes. Simulation was expanded upon from RRest software(https://github.com/peterhcharlton/RRest).<br><br>DeepBeat can be found at: https://github.com/AshleyLab/DeepBeat

For manuscripts utilizing custom algorithms or software that are central to the research but not yet described in published literature, software must be made available to editors and reviewers. We strongly encourage code deposition in a community repository (e.g. GitHub). See the Nature Research guidelines for submitting code & software for further information.

Data

Policy information about availability of data

All manuscripts must include a data availability statement. This statement should provide the following information, where applicable:

- Accession codes, unique identifiers, or web links for publicly available datasets
- A list of figures that have associated raw data
- A description of any restrictions on data availability

The data used to train DeepBeat (train, validate and test partitions) is available through synapse (Synapse ID:syn21985690). Ambulatory dataset was made available to Stanford for the current study, and is not publicly available.

April 2020

Field-specific reporting

Please select the one below that is the best fit for your research. If you are not sure, read the appropriate sections before making your selection.

☒ Life sciences ☐ Behavioural & social sciences ☐ Ecological, evolutionary & environmental sciences

For a reference copy of the document with all sections, see nature.com/documents/nr-reporting-summary-flat.pdf

Life sciences study design

All studies must disclose on these points even when the disclosure is negative.

Sample size	We simulated over 1 million PPG signals for pretraining the DeepBeat model. For fine-tuning, the following studies were used: Cardioversion cohort(n=107), Exercise stress test cohort (n=41), Ambulatory Cohort (n=15) and pulse oximetry (n=42)
Data exclusions	We considered only individuals with a clinically documented AF episode, all other arrhythmias where excluded.
Replication	In order to verify reproducibility we choose to included two external validation datasets. One that consisted of ambulatory monitoring and a second, publicly available non-AF dataset. We tested our model on these external datasets for reproducibility of our model results. In addition we have included a notebook on our GitHub to reproducible the testing results of our trained model.
Randomization	The data were randomly allocated to the training, validation and testing sets.
Blinding	Blinding was not needed for this study, in order to successfully train our model we needed to know the true labels of the dataset used.

Reporting for specific materials, systems and methods

We require information from authors about some types of materials, experimental systems and methods used in many studies. Here, indicate whether each material, system or method listed is relevant to your study. If you are not sure if a list item applies to your research, read the appropriate section before selecting a response.

Materials & experimental systems

n/a	Involved in the study	
☒	☐	Antibodies
☒	☐	Eukaryotic cell lines
☒	☐	Palaeontology and archaeology
☒	☐	Animals and other organisms
☐	☒	Human research participants
☒	☐	Clinical data
☒	☐	Dual use research of concern

Methods

n/a	Involved in the study	
☒	☐	ChIP-seq
☒	☐	Flow cytometry
☒	☐	MRI-based neuroimaging

Human research participants

Policy information about studies involving human research participants

Population characteristics	Population characteristics can be found in Table 1 of our paper.
Recruitment	Patients self-selected into our study.
Ethics oversight	Institutional Review Board of Stanford University

Note that full information on the approval of the study protocol must also be provided in the manuscript.
